# Thyroglobulin and thyroglobulin antibodies in differentiated thyroid cancer: interpretation, challenges, and future perspectives

**DOI:** 10.1530/JME-25-0217

**Published:** 2026-05-15

**Authors:** M H Links, T P Links, W T Zandee, A C Muller Kobold

**Affiliations:** ^1^ Department of Endocrinology, University of Groningen, University Medical Centre Groningen, Groningen, The Netherlands; ^2^ Department of Laboratory Medicine, University of Groningen, University Medical Centre Groningen, Groningen, The Netherlands

**Keywords:** thyroid cancer, thyroglobulin, thyroglobulin antibodies, clinical interpretation, congenital hypothyroidism

## Abstract

Thyroglobulin (Tg) is a thyroid-specific protein playing a key role in thyroid hormone production. For patients with differentiated thyroid carcinoma (DTC), Tg is the main biomarker during follow-up determining treatment response and risk of disease recurrence. Accurate Tg measurement is complicated by assay-specific performance and interference, most notably thyroglobulin autoantibodies (TgAbs). High-sensitivity Tg assays can detect very low Tg levels, eliminating the need for TSH-stimulated Tg measurements. Because of potential TgAb interference in Tg assays, TgAb measurement must always accompany Tg analysis. TgAbs are present in a minority of healthy individuals, but in up to one-third of patients with DTC. While low-level TgAbs are often clinically irrelevant, higher titres interfere with Tg immunoassays, possibly causing false-negative Tg results. Importantly, TgAb trends themselves provide prognostic information: declining concentrations support remission, whereas rising titres may indicate recurrence, offering a potential useful surrogate marker when Tg is unreliable. Alternative and complementary laboratory strategies are emerging. Novel proteomic approaches may allow isoform-specific Tg detection, potentially distinguishing tumour-derived Tg from normal Tg. In parallel, liquid biopsy technologies, particularly cell-free DNA, circulating tumour DNA, and microRNA assays, are being explored for non-invasive detection of minimal residual disease, although their role in DTC remains to be defined. In summary, Tg and TgAbs remain the mainstay of laboratory monitoring in DTC. Their interpretation demands awareness of assay limitations, appropriate cut-offs, and the clinical context. Future integration of advanced proteomic and genomic biomarkers holds promise for more specific, individualized surveillance strategies in thyroid cancer.

## Thyroid carcinoma: general background

Thyroid carcinoma is a relatively uncommon malignancy, but its incidence has steadily increased from 2.01 to 2.83 per 100,000 population globally between 1990 and 2019 (1). The rise in incidence in contrast to stable mortality numbers seems to point out overdiagnosis of indolent thyroid cancers contributing to overtreatment and societal disease burden (1). Women are more frequently diagnosed than men at a median age of approximately 50 and 55 years, respectively (2). Thyroid carcinoma can be divided into differentiated thyroid carcinoma (DTC), comprising papillary (PTC), follicular (FTC), and oncocytic thyroid carcinoma (OTC; formerly Hürthle cell carcinoma), and accounts for approximately 80–90% of thyroid cancer cases. Less common are medullary thyroid carcinoma (MTC), derived from parafollicular C-cells, and anaplastic thyroid carcinoma (ATC), an undifferentiated subtype with a very poor prognosis.

Prognosis is strongly determined by histological subtype and stage at diagnosis. Patients with DTC generally have good prognosis, with 5-year survival rates around 98% (2). In contrast, MTC carries an intermediate prognosis with 5-year survival of 93%, while ATC has median survival of only a few months and a 10% 5-year survival rate (2). Risk stratification frameworks, such as the American Joint Committee on Cancer/Union for International Cancer Control (AJCC/UICC) tumour–node–metastasis (TNM) staging, American Thyroid Association (ATA), and European Society for Medical Oncology (ESMO) risk classifications, are used internationally to guide treatment and follow-up while emphasizing a tailored approach based on patient risk (3, 4, 5).

According to the ATA and ESMO guidelines, treatment is tailored to risk: patients with low-risk DTC (for example, tumours 1–4 cm without aggressive features) may be managed with lobectomy and ultrasound-based follow-up, while patients with higher-risk DTC usually undergo total thyroidectomy and possibly radioactive iodine (RAI) therapy (3, 5). For DTC, serum thyroglobulin (Tg) and antibodies against thyroglobulin (TgAbs) are key biomarkers in diagnostics and follow-up. Their clinical utility and limitations will be discussed in detail in subsequent sections of this review.

## Thyroglobulin: biology, variants, and clinical relevance

**Figure 1:**
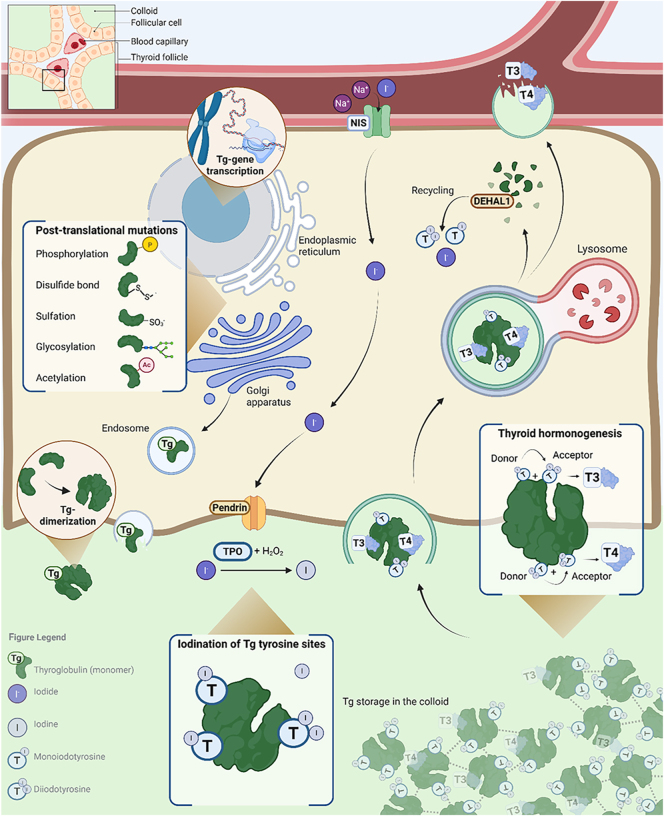
Thyroglobulin formation, post-translational mutations, storage, and role in thyroid hormonogenesis. After gene transcription, the Tg monomer is formed in the endoplasmic reticulum (ER). Post-translational mutations occur in the ER and Golgi apparatus, and Tg is brought to the intrafollicular space where it dimerized and became iodinated at available tyrosine sites. Thyroid hormonogenesis occurs at particular hormonogenic sites where donor–acceptor interactions can take place. Tg is stored in the colloid until endosomal uptake into the thyroid follicle. Here, the T3 and T4 hormones are spliced from Tg and transported into the bloodstream. The Tg protein is further degraded, but iodide and mono- and diiodotyrosines are recycled by DEHAL1. Created in BioRender by Links M (2026): https://BioRender.com/f9ahhbq.

Tg is a large dimeric glycoprotein produced by thyroid follicular cells and serves as the scaffold and storage matrix for iodide and thyroid hormone synthesis (6). The human Tg gene maps to chromosome 8q24, and its genetic code is moderately conserved over different species; sequence identity is approximately 48% (Xenopus), 43% (zebrafish), and 31% (lamprey) compared to human and consistent with conserved function (7). Tg monomers are initially formed in the endoplasmic reticulum (ER; Fig. 1), where Tg messenger RNA codes for a polypeptide of 2,750–2,760 amino acids (330 kDa). Second, the Tg monomers undergo many post-translational modifications (PTMs) in the ER and Golgi apparatus, which have a major impact on Tg function and signalling as they influence folding, local charge, solubility, stability, oligomerization and affinity for binding other molecules (8, 9). Tg is a multidomain protein consisting of different regions, including the N-terminal, which is composed of cysteine-rich repeated motifs allowing formation of disulphate bonds, and the C-terminal, which is rich in tyrosine and contains a cholinesterase-like domain likely facilitating intracellular transport of Tg (6, 8). After formation, folding, and undergoing the necessary PTMs, Tg finally matures after its excretion in the follicular lumen, where two monomers combine to form a 660 kDa homodimer. In the follicular lumen, the Tg dimers cross-link to form the colloid. Long-term storage of Tg in high densities possibly occurs through phase separation, a process leading to transition of normally very soluble proteins to liquid- or gel-like condensates without altering chemical bonds (10).

Tg plays a key role in iodine storage and synthesis of thyroid hormones (T3 and T4) by coupling iodine to available tyrosine sites on the Tg protein. Iodide (I^−^; the unoxidized form of iodine) is an essential micronutrient transported from the blood into the thyroid follicular cells by the natrium/iodide symporter (NIS) and passively migrated into the extrafollicular colloid by pendrin where its oxidation is facilitated by thyroid peroxidase (TPO) and H_2_O_2_ generated by dual oxidase (DUOX) (11, 12). This allows binding of iodine to Tg tyrosine sites, resulting in the formation of monoiodotyrosine (MIT) or diiodotyrosine (DIT). Thyroid hormones are formed after the combination of MIT and/or DIT through a donor/acceptor interaction resulting in triiodothyronine (T3; MIT + DIT) or tetraiodothyronine (also thyroxine, T4; DIT + DIT) at the acceptor site and leaving a dehydroalanine (Dha) at the donor site (8). While Tg contains a total of four hormonogenic sites where donor/acceptor interactions take place, the main thyroid hormone synthesis occurs at the N-terminus, mainly producing T4, and the C-terminus, mainly forming T3 (13). Each Tg dimer contains around 66 tyrosine-binding sites from which (depending on iodine status) around 30 are iodinated, resulting in an average of 10–15 MITs and DITs allowing formation of a maximum of seven thyroid hormone molecules at the four donor/acceptor sites (6, 13). After the formation of thyroid hormones, Tg can remain in the colloid for storage until endocytic uptake into the thyroid follicle where T3 and T4 are cleaved from Tg by cathepsin-mediated lysosomes and excreted in the bloodstream (8). Remaining MITs and DITs on the Tg protein are recycled into iodide and tyrosine by thyroid dehalogenase (DEHAL1) to be reused, and remaining Tg fragments are further degraded into amino acids (14). Thyroid-stimulating hormone (TSH) regulates all steps in the Tg cycle from Tg transcription/export to iodide uptake via NIS and Tg endocytosis/proteolysis for thyroid hormone release (6, 13).

### Tg isoforms and structural heterogeneity

Across the spectrum of thyroid diseases, multiple isoforms of thyroglobulin (Tg) exist, primarily caused by differences in post-translational modifications (PTMs), by proteolytic processing, and to a lesser extent through alternative splicing or common coding SNP variants. PTMs are part of the normal formation of Tg and occur mainly in the ER and Golgi apparatus (9). The predominant PTMs include N-glycosylation, disulphide bond formation, and variable iodination, while less frequent but well-documented modifications comprise phosphorylation, sulphation, and acetylation (8). These modifications regulate Tg folding, intracellular transport, hormone synthesis site accessibility, and epitope exposure, thereby shaping both its biological function and immunogenic properties. Specific mutations in the Tg gene or adaptation of PTMs may result in incorrect formation or folding of the Tg protein, leaving Tg trapped in the ER and afunctional, causing congenital hypothyroidism (15). A recent preprinted study identified several Tg missense variants associated with hypothyroidism in the United States population (16). Their results suggest that certain Tg isoforms might cause a more subtle or late-onset hypothyroidism than previously recognized.

Emerging work suggests that in autoimmune thyroid diseases, PTMs (importantly iodination) can generate neo-epitopes on Tg that escape central tolerance and are likely to contribute to the immunopathogenic TgAb responses (17, 18). Other isoforms of Tg originate from genetic polymorphisms and splice variants. A notable example is a monogenic form of Hashimoto’s thyroiditis caused by a splice site mutation (c.1076-1G>C) leading to exon 9 skipping and a structurally altered Tg protein (19). Single nucleotide polymorphisms (SNPs) across various Tg exons (e.g. exons 10, 12, and 33) have been associated with autoimmune thyroid diseases (AITDs) in different ethnic populations, including Chinese, Japanese, and Caucasians (20, 21, 22). Importantly, polymorphisms in intron 41 have shown strong association with AITDs in the Japanese population, suggesting that this intronic region may affect Tg expression or processing in a disease-relevant way (23). Conversely, these associations could not be reproduced in certain populations (e.g. Tunisians and United Kingdom), where studies showed no significant association between Tg SNPs and AITDs, indicating genetic heterogeneity across ethnic groups (24, 25).

In thyroid cancers, gene mutations or altered PTMs might also generate Tg isoforms. Several studies have identified hypoiodinated Tg in DTC possibly resulting from non- or poor formation of thyroid follicles by tumour cells, preventing normal Tg iodination (26). Other PTMs in tumour Tg, particularly changes in glycosylation patterns, have been suggested to contribute to carcinogenesis and antigenicity (9). Future advances in structural Tg analysis might allow distinction of tumour-derived Tg from normal-tissue derived Tg, enhancing diagnosis and follow-up of thyroid cancer.

In summary, Tg is a large dimeric glycoprotein produced by thyroid follicular cells and plays a key role in thyroid hormone production and iodine storage. After formation, Tg monomers undergo many post-translational modifications (PTMs) that play a major role in determining Tg function and characteristics. After transport into the follicular lumen, Tg monomers combine to form the Tg dimer, which becomes heavily iodinated at its many available tyrosine sites. Tg contains four hormonogenic sites allowing combination of monoiodotyrosine and diiodotyrosine to form triiodothyronine (T3) or tetra-iodothyronine (T4). To release thyroid hormones, Tg is taken up into the thyroid follicle and cleaved, allowing T3 and T4 to be transported to the bloodstream. Mistakes in the process of Tg formation or PTMs can lead to afunctional Tg and cause hypothyroidism or might predispose an individual to autoimmune thyroid diseases.

## Clinical value of Tg in differentiated thyroid cancer

In patients with DTC, Tg serves as a crucial biomarker during follow-up to determine treatment response and risk of disease recurrence (Table 1). The initial risk of recurrence is estimated after surgery by tumour type (PTC, FTC, and OTC), TNM stage, and histological tumour characteristics. While the 2015 ATA guidelines used a three-tier system to predict the initial risk of structural recurrence (high, intermediate, and low risk), the newer 2025 ATA guidelines introduce a four-tier system to estimate the risk separately for PTC, FTC, and OTC (3). Using this system, the initial risk of recurrence is divided into low (<10%), low-intermediate (10–15%), high-intermediate (≥16–30%), and high (>30%). Monitoring Tg levels after treatment allows evaluation of treatment response and determines the ongoing risk stratification. This ongoing risk stratification (Tg values over time), used in combination with the initial risk class assignment (histopathological tumour features), allows a more precise and dynamic prediction of persistent or recurrent disease and a tool for the clinician to provide individualized treatment recommendations.

**Table 1:** European (ESMO) and American (ATA) guideline recommendations for excellent, indeterminate, and biochemically incomplete treatment responses with thyroglobulin and thyroglobulin antibodies.

Treatment response	Treatment received	ESMO (2019) (5)	ATA (2025) (3)
Excellent	Post-total thyroidectomy ± neck dissection and RAI	Non-stimulated-Tg < 0.2 ng/mL or stimulated-Tg < 1 ng/mL	
	Post-total thyroidectomy ± neck dissection	Non-stimulated-Tg < 0.2 ng/mL	Non-stimulated-Tg < 2.5 ng/mL
	Post-lobectomy	Undetectable TgAb and stable Tg	Normal, low-risk, or benign biopsy nodules in the contralateral lobe
Indeterminate	Post-total thyroidectomy ± neck dissection and RAI	Non-stimulated-Tg 0.2–1 ng/mL or stimulated-Tg 1–10 ng/mL or TgAb stable/declining	
	Post-total thyroidectomy ± neck dissection	Non-stimulated Tg 0.2–5 ng/mL or TgAb stable/declining and negative imaging	Non-stimulated Tg 2.5–5 ng/mL or TgAb stable/declining and negative imaging
	Post-lobectomy	Non-specific imaging findings	-
Biochemically incomplete	Post-total thyroidectomy ± neck dissection and RAI	Non-stimulated-Tg > 1 ng/mL or stimulated-Tg > 10 ng/mL or increasing TgAb	
	Post-total thyroidectomy ± neck dissection	Non-stimulated-Tg > 5 ng/mL or increasing TgAb	
	Post-lobectomy	Increasing Tg at stable TSH values or rising TgAb levels	-

ESMO, European Society for Medical Oncology; ATA, American Thyroid Association; RAI, radioactive iodine; Tg, thyroglobulin; TgAb, thyroglobulin antibodies; TSH, thyroid-stimulating hormone.

### Stimulated or unstimulated Tg measurements

After a total thyroidectomy, Tg values have been instrumental in both early detection and long-term surveillance. Previously, measurement of stimulated Tg by either THW or rhTSH was recommended, allowing a more accurate estimation of residual disease when using the older Tg assays. However, with the introduction of high-sensitivity (hs)-Tg assays, reliance on stimulated measurements has decreased as stimulated and unstimulated measurements are highly correlated (27). Several studies have demonstrated the clinical utility of hs-Tg. A meta-analysis reported a negative predictive value of 97–99% for recurrence when Tg measured after a total thyroidectomy with RAI therapy during levothyroxine therapy was <0.1 ng/mL (28). Groen *et al.* demonstrated that a hs-Tg measurement three months after ablation predicted which patients would also have undetectable Tg after thyroid hormone withdrawal six months later, with a 97.7% negative predictive value (29). Thus, in DTC patients after a total thyroidectomy and RAI, undetectable hs-Tg combined with a negative ultrasound obviates the need for stimulated Tg testing.

### Tg value and risk of recurrence

Response to DTC treatment is categorized into excellent, indeterminate, or biochemically incomplete mainly based on Tg values (Table 1). After achieving an excellent response, the risk of disease recurrence depends on the initial risk classification: in patients with an initial low-risk DTC-classification, recurrence rates are <2%. However, in patients with a high initial risk assignment, recurrence rates up to 30% have been reported despite achieving an excellent response (30). Cut-off values for Tg in these treatment-response categories depend on the type of treatment received. When a total thyroidectomy is followed up with RAI, a non-stimulated Tg < 0.2 ng/mL measured with a hs-Tg assay is generally regarded as an excellent response (3). After a total thyroidectomy without RAI ablation, the presence of remaining microscopic thyroid tissue may result in detectable Tg without tumour recurrence, resulting in a higher Tg cut-off for recurrent disease in this patient group. In an earlier systematic review, non-stimulated Tg values from <2.5 ng/mL suggested a low risk of recurrence or metastatic disease with a median sensitivity of 0.93 and specificity of 0.48 (31). Yet, the available studies together provide a low strength of evidence (31). Nevertheless, this cut-off of 2.5 ng/mL is incorporated in the treatment response evaluation of the recent ATA guidelines with non-stimulated Tg < 2.5 ng/mL after a total thyroidectomy without RAI, indicating an excellent treatment response (3). The ESMO guidelines use a stricter Tg cut-off below 0.2 for an excellent treatment response after a total thyroidectomy without RAI (5). A very recent retrospective study from Korea showed the prognostic value of these moderately increased Tg values (0.3–2.5 ng/mL) for this patient group demonstrating stepwise increases in 10-year recurrence risks with increasing Tg levels, with risks of 0.5% for Tg < 0.3 ng/mL, 2.6%–3.2% for Tg between 0.3 and 2.5 ng/mL, and 7.1% for Tg levels 2.5–5.0 ng/mL (32). While the cut-off of 0.3 ng/mL yielded the highest sensitivity (83.8%) and specificity (74.3%) in this large cohort, the 10-year risk of recurrence for a Tg cut-off <2.5 ng/mL remained below 4% and therefore still fits in the ATA 2025 excellent response group, where the mean recurrence is 1–4%. Finally, any rise in Tg over time, even from initially low or undetectable Tg levels in this excellent response group, is associated with an increased risk of DTC recurrence and warrants further investigation (3, 5, 32).

A ‘grey zone’ of Tg values (typically 0.2–1 ng/mL in patients who underwent a total thyroidectomy with RAI or 2.5–5 ng/mL in patients who underwent a total thyroidectomy alone) during TSH suppression is associated with an increased risk of structural recurrence. However, during follow-up, Tg still decreases in a large proportion of patients and, therefore, this is regarded as an indeterminate response to therapy (33). In this group, recurrence rates range from 5 to 20% (3). A stimulated Tg might be useful in patients with an indeterminate response as a low stimulated Tg is associated with a low risk of recurrence (34). However, stimulated Tg comes at the expense of hypothyroidism (THW) or higher financial costs (rhTSH). Moreover, Tg results may vary with the timing of sampling relative to the TSH peak (27). In the end, since the stimulated Tg measurements rarely change clinical management, continued serial Tg monitoring (per 6–12 months) is generally recommended for patients with an indeterminate response to treatment.

A Tg under TSH suppression therapy persistently above 1 ng/mL after a total thyroidectomy with RAI, or above 5 ng/mL after a total thyroidectomy alone, represents a biochemically incomplete response to therapy and warrants closer evaluation, particularly if Tg levels are rising over time. Recurrence rates in case of an isolated biochemically incomplete treatment response are around 20–53% but become 85% in studies combining biochemically and structurally incomplete treatment responses (3). Even small but consistent increases are more concerning than a single isolated higher value, underscoring the importance of longitudinal follow-up using the same assay. In those cases, further evaluation typically begins with neck ultrasound, followed by ^18^FDG-PET/CT if indicated (3).

A lobectomy is often performed in patients with a low-risk DTC (e.g. unilateral cT1-2 DTC without high-risk features). After a lobectomy, the remaining thyroid tissue still contributes to the serum Tg pool and this lobe might even show compensatory growth, causing a gradual increase in Tg in patients without structural recurrent disease (35). A recent meta-analysis showed a major heterogeneity in Tg values after a lobectomy in low-risk patients, making Tg unsuitable to detect recurrent disease in these patients (36). While current ESMO guidelines include Tg and TgAbs in their risk stratification after a lobectomy, there is at present not enough evidence of their reliability as biomarkers in this setting and, therefore, for these patients, follow-up with ultrasound should be preferred, in line with the recent ATA guidelines (3, 5).

### Clinical implication of TgAb for risk of recurrence

The presence of autoantibodies against Tg (TgAbs) in serum is a well-known interfering factor for current Tg assays potentially leading to an underestimation of Tg and failure to detect DTC recurrence. For this purpose, Tg must always be measured in combination with TgAb to identify possible interference. However, TgAbs are not solely interfering factors as changes in TgAb levels over time have shown promise as a predictive marker for disease progression and response to treatment in DTC patients. These changes in TgAb levels likely reflect an immunological response to the presence of thyroid (cancer) tissue. Several longitudinal studies, including our own, show that falling TgAb titres after surgery and ablation correlate with remission, while rising titres often are associated with an increased risk of recurrence (37, 38, 39). In these studies, patients who converted from TgAb-negative to TgAb-positive or showed ≥50% rises were significantly more likely to have persistent or recurrent disease, whereas those whose TgAb titres declined ≥50% almost invariably achieved remission. This dynamic behaviour makes TgAb trends valuable in guiding follow-up intensity, particularly when Tg cannot be relied upon. However, as absolute TgAb quantity does not correspond to tumour load, TgAbs should be interpreted in time (40).

### Tg measurements in fine-needle lymph node aspirates (FNA)

Tg measurement has also been extended to other matrices than blood, such as fine-needle aspiration washout (FNA-Tg) from cervical lymph nodes. Positive FNA-Tg strongly supports metastatic DTC even when cytology and radiology are inconclusive (41). False positives, however, do occur, especially in the lymph nodes of the central compartment when the thyroid gland is still present (42). Reported cut-offs vary between assays and sampling protocols and depend on whether thyroid tissue remains *in situ*, but diagnostic accuracy is consistently high (43). On interpretation, one must consider possible contamination with blood and the presence of TgAbs.

### Tg in other thyroid tumours

Tg is not a tumour marker for all follicular cell-derived thyroid cancers. In poorly differentiated thyroid carcinoma or anaplastic thyroid carcinoma, Tg gene expression is often decreased due to dedifferentiation and TgAbs are rarely induced (44). In these tumours, tumour-derived Tg is often not measurable in blood serum. Hypothetically, these poorly differentiated tumours could possibly secrete severely altered Tg, due to mutations or PTMs, making them undetectable with current Tg assays given the changes in folding or Tg epitopes. New techniques, such as electron microscopy (cryo-EM, allowing structural analyses of Tg), might identify these differences in Tg folding and surface properties, which could in the future allow investigation of more heterogeneous Tg in these poorly differentiated tumours.

In summary, Tg serves as a crucial biomarker in the follow-up of DTC. The need for TSH-stimulated Tg has minimized with the introduction of hs-Tg assays as the stimulated and unstimulated measurements are highly correlated. Post-therapy Tg levels determine treatment response (excellent, indeterminate, or biochemically incomplete) depending on the initial treatment (total thyroidectomy with/without RAI) and predict risk of tumour recurrence. After a lobectomy, Tg interpretation is limited by the presence of normal thyroid tissue and therefore has a low sensitivity for detection of recurrence. When TgAbs are present in serum and interfere with Tg measurements, trends in TgAbs have shown to serve as a predictive marker for disease progression or recurrence. Tg measured in lymph node aspirations strongly suggests lymph node metastases. In other types of thyroid cancer, Tg is not useful as a tumour marker as these secrete no (detectable) Tg.

## Thyroglobulin assays

**Figure 2:**
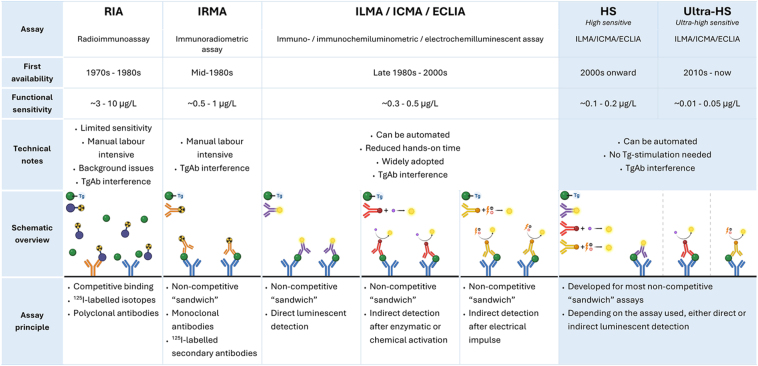
Evolution, sensitivity, and key characteristics of thyroglobulin immunoassays (IMAs). Images in this table are created in BioRender by Links M (2026) – RIA: https://BioRender.com/ifuj2uz; IRMA: https://BioRender.com/7ag2a35; ILMA: https://BioRender.com/9bhvwq7; ICMA: https://BioRender.com/75u04pg; ECLIA: https://BioRender.com/d515x2z; HS/ultra-HS: https://BioRender.com/3z6r8q7.

The first Tg assay was introduced in the 1970s, and since then, assay technology has evolved considerably, each generation addressing earlier shortcomings (Fig. 2).

### Radioimmunoassays

The earliest assays were competitive radioimmunoassays (RIAs), in which radiolabelled Tg competed with patient Tg for antibody binding. These assays, relying on polyclonal antibodies, were limited by poor specificity and relatively high functional sensitivities ranging from 3 to 10 ng/mL. While possibly less affected by assay interferences compared to some of the later developed techniques, the use of polyclonal antibodies increases the risk of cross-reactivity, and the level of sensitivity is inadequate for detecting minimal residual disease.

### Immunometric assays

The development of monoclonal antibodies and the non-competitive ‘sandwich’ format led to the use of immunometric assays (IMAs) for Tg. At first, the immunoradiometric assays (IRMAs) were introduced, and afterwards, IMAs without radioactive labelling were developed, such as immunoluminometric, immunochemiluminescent, and electrochemiluminescent immunometric assays (ILMAs, ICMAs, and ECLIAs; Fig. 2). These newer assays achieved functional sensitivities of 0.3–0.5 ng/mL, allowing confident detection of Tg at sub-ng/mL levels. Since then, the so-called high-sensitive Tg (hs-Tg) assays or even ultra-sensitive Tg assays have further improved follow-up by reliably detecting Tg concentrations well below 0.1 ng/mL, enabling Tg monitoring during thyroid hormone replacement (suppressed TSH), without the need for routine TSH stimulation (45).

Despite calibration of Tg-IMA assays against the BCR457-certified reference material, assays still differ in the antibodies they use, recognizing different epitopes and isoforms of Tg. In fact, when using Tg-IMAs from different manufacturers and TgAb-negative samples, Tg results differed significantly with a coefficient of variability (CV) of 37% (46). This variability has clinical consequences and needs to be considered when interpreting results from other laboratories. Inter-method variability is also high with a CV of around 30% comparing IMA, to RIA and LC–MS/MS in TgAb-negative serum (47). Therefore, numerical Tg results from different assays and methods cannot reliably be compared, emphasizing the importance of using the same Tg assay during follow-up of DTC patients.

### Liquid chromatography–tandem mass spectrometry

**Figure 3:**
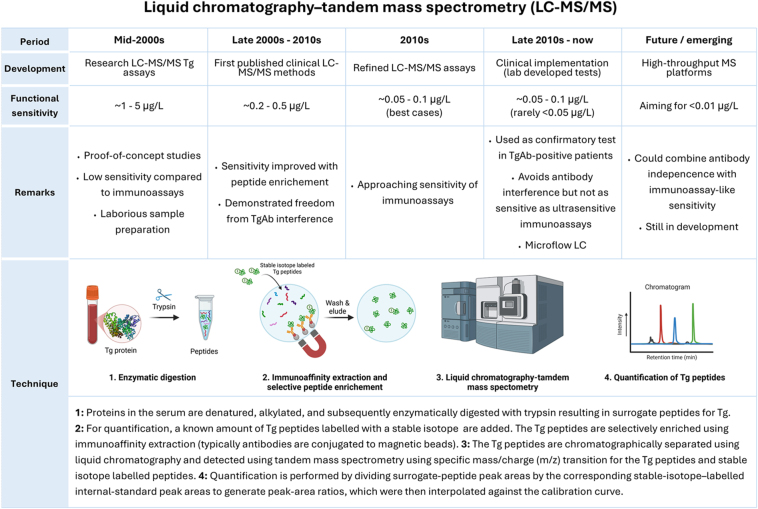
Developments in liquid chromatography–tandem mass spectrometry (LC–MS/MS) for measuring Tg. Images in this table are created in BioRender by Links M (2026): https://BioRender.com/kcldb65*.*

In addition to immunoassays, liquid chromatography–tandem mass spectrometry (LC–MS/MS) has been introduced as an alternative approach (Fig. 3). By quantifying Tg-derived peptides after proteolytic digestion, LC–MS/MS is largely unaffected by TgAbs and heterophile antibodies, making it valuable as a confirmatory tool in suspected interference. However, Tg by LC–MS/MS has been reported to be lower than expected in TgAb-positive patients possibly due to (*in vivo*) biological clearance of Tg–TgAb-bound immune complexes (48). These immune complexes might be cleared more rapidly by the reticuloendothelial system, genuinely reducing the total circulating Tg pool. This ‘immune-complex clearance’ is a well-known immunological phenomenon and is discussed in the Tg/TgAb literature (9). As this immune-complex clearance is not an assay artefact, this phenomenon would affect not only LC–MS/MS assays, but all other assays in a same way.

### Pre-analytical influencing factors and analytical interferences of Tg

Pre-analytical factors may contribute to Tg variability. Age, sex, iodine status, smoking, and TSH-drive all influence baseline Tg levels. In the first 2 years of life, Tg levels are usually highest, generally attributed to the TSH surge in early development (49). Especially in neonates, a sharp rise in Tg levels occurs in the first days of life, most pronounced in preterm infants and those with respiratory distress syndrome independent of the TSH surge, suggesting impaired Tg degradation or haemoconcentration (50). Cord-blood Tg concentrations are further influenced by delivery mode and are significantly higher in infants of smoking mothers, likely due to thiocyanate-mediated fetal thyroid effects (51, 52). From a laboratory perspective, Tg and TgAbs are generally stable during routine handling, even after few freeze–thaw cycles, but prolonged storage (years not months) may influence measured concentrations (53).

Despite analytical advances, Tg measurement is vulnerable to interferences, which can mask epitopes and cause falsely low or falsely high results depending on the assay type used. Among these interferences, TgAbs are most clinically and analytically relevant and will be elaborated on in the next paragraph. The high-dose hook effect is another analytical interference, which, even in modern assays, creates false-low results in samples with extremely high Tg concentrations. This effect occurs when a surplus of Tg binds all the available detection antibodies, preventing formation of the sandwich complex and resulting in spuriously low measured Tg values unless repeat testing with dilution is performed (54). Although uncommon (0.3–3%), Tg assays can also be affected by the presence of heterophile antibodies and human anti-mouse antibodies (HAMAs; formed after exposure to mice protein, e.g. after treatment with biologicals), which can cross-link assay antibodies and cause falsely high or low Tg results (55). Finally, biotin (known as vitamin B_7_ or vitamin H) can interfere with biotin–streptavidin-based Tg assays and, depending on the assay, lead to falsely low or falsely high Tg values. Over-the-counter use of biotin is increasingly common, and interfering biotin levels (≥10 ng/mL) were found in 7.4% of 1,142 patients presenting to a United States emergency department (56). In general, because such mismatches may only become apparent in the clinical context, they may go unnoticed in the laboratory despite comprehensive quality checks. These assay interferences, however, are often first suspected by the clinician when laboratory results do not fit the patient’s clinical presentation, underscoring the importance of close communication between the treating clinician and the laboratory.

In summary, Tg assay technology has advanced from insensitive competitive RIAs to high-sensitive IMAs, complemented by LC–MS/MS. hs-Tg assays have transformed clinical practice by enabling reliable Tg monitoring without the need for TSH stimulation. In general, assay interferences are often first suspected by the clinician when laboratory results do not fit the patient’s clinical presentation, underscoring the importance of close communication between the treating clinician and the laboratory. Yet, awareness of assay limitations, timing, and potential interferences remains essential to ensure accurate interpretation and optimal patient care.

## Antibodies against thyroglobulin (TgAbs)

TgAbs occur in a wide spectrum of thyroid disorders and in the general population, but their frequency varies markedly in different populations. In healthy individuals, TgAbs are detectable in roughly 8–13% (57, 58), whereas in differentiated thyroid carcinoma (DTC) cohorts, the prevalence rises to 18–29% or even 41% in paediatric DTC (59, 60, 61, 62, 63). In autoimmune thyroid disease, particularly Hashimoto’s thyroiditis, TgAbs are present in 80–90% of patients and are often accompanied by antibodies to thyroid peroxidase (TPO), whereas in Graves’ disease, somewhat lower percentages (30–60%) are found (64). Thus, TgAb positivity in DTC patients reflects both overlap with autoimmune thyroid disease and a possible tumour-related immune response. The reason TgAbs are more common in DTC and autoimmune thyroid diseases than in healthy controls is not fully understood. A prevailing hypothesis is that the structural heterogeneity of Tg creates neo-epitopes that break immune tolerance (17, 18). Tg is a very large protein with hundreds of potential antigenic sites. Mapping studies show that TgAbs recognize conformational epitopes distributed across the molecule, with clusters near hormone synthesis sites. Hashimoto’s thyroiditis and DTC appear to generate partially overlapping but not identical epitope repertoires (8). Some data suggest that TgAbs in HT more often recognize epitopes exposed on highly iodinated Tg, while TgAbs in DTC may be directed against alternative or modified regions (8, 65). The precise link between Tg isoforms and TgAb specificity remains incompletely defined, but the epitope–isoform relationship is a promising field for LC–MS/MS-based investigation.

### TgAb assays

TgAb assays are heterogeneous, with per laboratory a different assay calibration and reporting units, resulting in poor concordance and high variability of TgAb results (46, 66). This could potentially affect the follow-up strategy as shown by a study comparing two TgAb assays and reporting disagreement in TgAb positivity in 11.4% of measurements (67). In October 2024, the World Health Organization’s Expert Committee on Biological Standardization (ECBS) introduced the first international standard for TgAb calibration, aiming to decrease variability. To our knowledge, there have not been comparisons of TgAb inter-assay variability since.

Before the routine availability of sensitive TgAb immunoassays, Tg recovery tests were widely used as a means of detecting antibody interference in Tg measurement. In these tests, a known quantity of labelled exogenous Tg is added (‘spiked’) to the patient’s serum. If the measured increase in Tg is markedly less than expected, the result is interpreted as evidence of interference, usually due to TgAbs binding to Tg and preventing its detection by the assay. While conceptually appealing, recovery tests have several major limitations: they are laborious, poorly standardized, and highly assay dependent. Discordant results between recovery tests and direct TgAb immunoassays are frequent, and a ‘normal’ recovery does not reliably exclude the presence of TgAbs (40). Several comparative studies and international guidelines have, therefore, concluded that recovery tests are neither sensitive nor specific enough to be clinically useful in the modern era (40, 68).

The current best practice is to measure TgAb directly with a validated immunoassay alongside every Tg determination and to interpret Tg in that context. TgAb measurement using an immunoassay is faster, more reliable, and more reproducible than recovery testing. Modern TgAb assays are automated immunometric assays, using chemiluminescent or electrochemiluminescent detection. They primarily detect IgG-class TgAbs, the clinically dominant isotype, although IgM and IgA antibodies may also occur at low levels. The antigen used is generally purified or recombinant human Tg, but differences exist in whether intact Tg, fragments, or partially denatured preparations are applied. As a result, TgAbs recognizing certain conformational or isoform-specific epitopes may be more or less detectable depending on the assay (67, 69). This explains why the same serum may test ‘positive’ in one assay but ‘negative’ in another and why absolute values differ by orders of magnitude across platforms.

### Defining TgAb positivity and implications for clinical relevance

One of the most debated issues is where to set the cut-off for TgAb positivity. Manufacturers typically define positivity at the manufacturer’s cut-off (MCO, Box 1), often derived from autoimmune thyroid disease cohorts. However, these cut-offs are thought to be too high to reliably flag antibodies that interfere with Tg immunoassays (40, 68, 69). In contrast, using the limit of detection (LoD) or functional sensitivity (FS) maximizes sensitivity but leads to nearly all DTC patients being labelled TgAb-positive, thereby rendering Tg unreliable in almost all patients (39).

Box 1 **Terminology of assay limits and cut-offs.**

**Table:** 

LoD	Limit of detection	Lowest concentration of analyte that can be distinguished from a blank with a chosen uncertainty; usually 2 standard deviations above the mean of 10 calibration replicates free of analyte
FS	Functional sensitivity	Lowest concentration of analyte that can be reliably measured with a coefficient of variation of 20%, sometimes also called limit of quantification
MCO	Manufacturer’s cut-off	Cut-off value as reported by the assay manufacturer
ICO	Institutional cut-off	Cut-off scores defined by each laboratory in their own population

**Figure 4:**
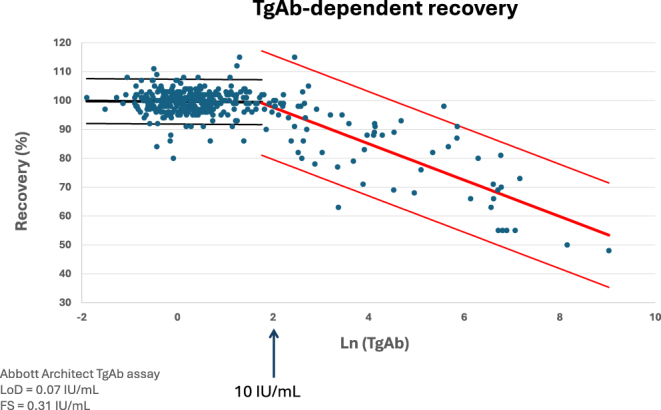
TgAb interference shown by a Tg recovery test. Unpublished data from the collaborative work of the VU University Medical Center, Amsterdam (VUMC, Heijboer), and the University Medical Center Groningen (UMCG, Klein-Hesselink and Muller Kobold). This figure illustrates the use of a Tg recovery test to indicate TgAb interference. The start of the downward slope signifies TgAb interference and corresponds in this case to TgAb levels of 10 IU/mL, corresponding to the UMCG institutional cut-off score determined according to the NACB guidelines.

Laboratories are advised to determine their institutional cut off (ICO) based on own techniques and patient population. The ICO can be determined using the National Academy of Clinical Biochemistry (NACB) guidelines by measuring TgAbs in 120 thyroid-disease-free participants (70). In addition to determining the ICO, one could argue that positive TgAbs should be defined as the level of clinically relevant TgAb interference with Tg assays. In this context, the Tg recovery test, although not reliable for detecting TgAb presence, does provide functional evidence of any active interference of TgAb with the Tg assay. In our institution, the ICO determined by the NACB guidelines described previously by Dekker *et al.* (39) corresponded with the TgAb cut-off using a Tg recovery test (both indicating a TgAb cut-off ≥10 IU/mL measured by Abbott Architect; Fig. 4), suggesting the feasibility of determining a clinically relevant ICO combining both methods.

We previously illustrated the differences in clinical relevance of the different cut-off values in a cohort of DTC patients for TgAbs measured by Abbott Architect; applying the LoD (0.07 IU/mL) or FS (0.31 IU/mL) made virtually all patients ‘positive’, eliminating Tg as a usable marker (39). Using the MCO (≥4.11 IU/mL) or an institutional cut-off (ICO, ≥10 IU/mL) identified 24 and 15% of patients as TgAb-positive, respectively. Importantly, TgAb positivity by MCO or ICO was not associated with tumour characteristics or risk profile, arguing against treating TgAb positivity as an independent risk factor. These findings are supported by other research groups, such as Latrofa and co-workers, who demonstrated that metastatic disease can be ruled out with TgAb below the MCO and undetectable Tg (71). Likewise, Côrtes *et al.* showed that low- or intermediate-risk DTC patients with TgAbs between the FS and MCO, undetectable Tg, and a normal neck ultrasound after initial treatment were not at greater risk of tumour persistence or recurrence compared to TgAb-negative patients (72).

A subsequent study performed by Dekker *et al.* confirmed that low-titre TgAbs (<10 IU/mL) are analytically detectable but clinically irrelevant: Tg measured by IRMA and LC–MS/MS, as an TgAb interference-free alternative method, agreed closely and outcomes did not differ (73). By contrast, higher-titre TgAbs (≥10 IU/mL) produced lower Tg values in immunoassays compared with LC–MS/MS, consistent with genuine interference. Thus, the ICO (in our institution, set at ≥10 IU/mL) seems more clinically meaningful than the LoD or FS for defining TgAb positivity in DTC follow-up. Difficulties in determining an ICO or borderline low-titre TgAbs could remain a reason for uncertainty in the interpretation of Tg results for both laboratorians and clinicians. In this case, longitudinal Tg and TgAb measurements remain the most conclusive indicators as a rise in either Tg or TgAb warrants further evaluation for disease recurrence.

In summary, TgAbs interfere with current Tg IMAs, potentially leading to false low-Tg results. Therefore, TgAbs should always be measured in parallel with Tg, using the same assay longitudinally. For DTC follow-up, low-titre TgAbs (between LoD and FS) are often analytically detectable but not clinically relevant; cut-offs set too low can cause unnecessary concern about interference. High-titre TgAbs (≥ICO, e.g. ≥10 IU/mL in our institute) are associated with meaningful Tg interference and justify interpreting Tg as reliable.

## Tg applications beyond DTC

Beyond differentiated thyroid carcinoma (DTC), Tg analyses may be useful in other applications, such as detection of congenital hypothyroidism or measuring an individual’s iodine status.

### Use of Tg in congenital hypothyroidism

Tg can play an important role in the post-screening diagnostic work-up of congenital hypothyroidism (74). After an abnormal heel prick suggestive of congenital hypothyroidism (CH), Tg can be measured in addition to TSH and free T4 to help distinguish between the major aetiological categories (Table 2). Because Tg is produced exclusively by thyroid follicular cells, its serum concentration reflects the presence and amount of functional thyroid tissue. Undetectable or very low Tg strongly suggests thyroid agenesis, while normal or elevated Tg indicates that thyroid tissue is present, supporting either ectopy or dyshormonogenesis as a leading diagnosis (74). The distinction is then made by ultrasound and scintigraphy. In rare cases, biallelic loss-of-function Tg mutations could also be the cause of CH, leading to dyshormonogenesis characterized by very low or undetectable serum Tg and a normal or enlarged thyroid on ultrasound. Overall, Tg can play a role in the assessment of CH as a biochemical indicator of thyroid tissue presence, location, and synthetic competence, guiding targeted imaging and genetic testing.

**Table 2:** Tg measurements for investigating congenital hypothyroidism aetiology.

Aetiology	Tg	Ultrasound	Scintigraphy	TSH	FT4	Typical clues
Thyroid agenesis	Low or undetectable	No gland	No uptake	Very high (often >50–100 mU/L)	Low	Very high TSH, persistent
Ectopic thyroid tissue	Low/normal/high	No gland in neck	Uptake ectopically	High	Low	Most common dysgenesis
Central hypothyroidism	Normal	Normal	Normal	Low, normal, or mildly elevated*	Low	Low/normal TSH + low FT4
Dyshormonogenesis	Normal/high	Normal or enlarged gland	Normal/high uptake	High (persistent)	Low	Often familial: several mutations in thyroid hormone synthesis
Tg gene defect	Low or undetectable	Normal or enlarged gland	Normal uptake	High	Low	Neonatal goitre

* Biologically inactive TSH is possible. This table shows a compact overview of aetiologies of congenital hypothyroidism.

Tg, thyroglobulin; TSH, thyroid-stimulating hormone; FT4, free T4.

### Use of Tg in the determination of iodine status

Tg in blood serum reflects both thyroid stimulation and iodine availability, making it a sensitive biochemical indicator of iodine status. Tg contains numerous iodination-competent tyrosine residues and serves as the intrathyroidal iodine storage. Under iodine deficiency, Tg becomes poorly iodinated, with reduced formation of MIT, DIT, and hormonogenic T4/T3 precursors, as shown by LC–MS/MS (65). Simultaneously, serum Tg concentrations rise due to TSH-driven thyroid hypertrophy, linking Tg changes directly to functional thyroid response. The World Health Organization (WHO) identifies Tg as a promising marker for monitoring iodine deficiency disorders, because serum Tg normalizes more rapidly after iodine repletion than thyroid volume or goitre rate, which reflect long-term iodine status (75). Population median Tg values < 13 μg/L in school-aged children indicate adequate iodine intake (76). Moreover, the possibility of measuring Tg levels from dried-blood spots makes this a feasible option even in low-resource countries (77). Tg measurement, including Tg iodination profiling, therefore offers complementary insight into both hormonogenesis and iodine status, strengthening evaluation of iodine nutrition and iodized-salt programmes.

In summary, Tg measurements can be used to investigate the aetiology of congenital hypothyroidism, where Tg levels can differentiate between the presence or absence of thyroid tissue. Moreover, Tg can be used to determine iodine status either directly by measuring iodination levels of Tg by LC–MS/MS, or a more available technique, or by determining serum Tg levels as an indirect reflection of iodine availability.

## Future laboratory perspectives in DTC: Tg isoform analysis, liquid biopsies, and multi-omics

Next-generation proteomic techniques, especially LC–MS/MS, offer increasing potential to differentiate between Tg isoforms derived from benign tissue and tumour tissue (65). This is especially relevant as lobectomy is now more frequently performed than total thyroidectomy. Moreover, identification of tumour-specific Tg isoforms may also allow development of more epitope-specific TgAb assays, especially relevant in Hashimoto’s thyroiditis versus DTC. This may improve the surrogate marker function of TgAbs by distinguishing those associated with autoimmunity from those signalling tumour‐related antigenicity.

Liquid biopsy approaches, measuring circulating tumour cells (CTCs), cell-free DNA (cfDNA), circulating tumour DNA (ctDNA), or exosomal microRNAs, are rapidly emerging across oncology. Early studies are promising for utilization of CTCs and cfDNA techniques (targeting DTC cells or several thyroid-specific genes, respectively) to detect tumour presence and disease progression, yet further technical optimization and standardization is required (78, 79). Advanced applications, such as analysis of cfDNA fragment patterns, methylation signatures, or cfDNA integrity measures, are actively under investigation in thyroid oncology (80). In parallel, ctDNA methods, such as targeted sequencing, enable detection of tumour-derived DNA and epigenetic markers with moderate to high sensitivity (78). Several miRNAs have been described showing high sensitivity and specificity for detection of thyroid cancer, with even better results when combined in a panel (81). These liquid biopsy techniques could improve non-invasive detection of minimal residual disease, clonal evolution, or early recurrence if specific mutations (e.g. BRAF and RAS) or fragmentomic patterns can be identified in DTC patients.

Emerging techniques allow combined analysis of proteomic (Tg isoforms), immunologic (TgAb epitope patterns), and genomic (cfDNA/ctDNA) biomarkers, using machine learning-driven integration and may yield highly specific and dynamic monitoring tools for DTC (82). Future DTC follow-up might be shaped by these multi-omics, which, possibly combined with hs-Tg and TgAb trends, could create individualized surveillance algorithms allowing early detection of disease activity.

## Discussion

Thyroglobulin and thyroglobulin antibodies remain the cornerstone in the follow-up of patients with differentiated thyroid carcinoma (DTC). Over the past decades, assay development has transformed their clinical utility: competitive RIAs with limited sensitivity have been replaced by high-sensitivity immunometric and/or LC–MS/MS assays capable of detecting Tg concentrations <1 ng/mL, thereby reducing the need for routine TSH stimulation. Parallel advances in TgAb assays have improved recognition of antibody interference, although significant variability persists between assays/platforms, particularly regarding cut-off definitions.

Several key lessons emerge. First, accurate interpretation of Tg results requires not only technical awareness of each assay’s functional sensitivity and susceptibility to interferences, but also strict adherence to longitudinal consistency in method use. TgAb measurement in parallel with Tg is still required, as antibodies remain the most relevant source of assay bias. Second, the clinical relevance of low-titre TgAbs is limited. Studies from our own group and others confirm that low-level TgAb positivity, while analytically detectable, rarely alters patient outcomes, underscoring the importance of using clinically meaningful cut-offs (e.g. institutional cut-offs rather than manufacturer’s thresholds). Third, TgAb dynamics provide additional information beyond interference as declining titres indicate remission, while rising titres often herald recurrence, making TgAb trends a useful surrogate marker of disease activity in TgAb-positive patients.

Despite these advances, challenges remain. Assay heterogeneity, both for Tg and for TgAb, continues to complicate cross-platform comparability. LC–MS/MS has emerged as an alternative method largely free of antibody interference, yet its current (financial and/or technical) availability limit widespread clinical adoption. Furthermore, interpretation of Tg in patients after lobectomy or in those with poorly differentiated or anaplastic carcinoma has little added value due to residual normal tissue or loss of Tg expression.

Looking ahead, the field is shifting towards increasing specificity and personalization. High-resolution proteomic approaches may enable detection of tumour-specific Tg isoforms or post-translational modifications, improving discrimination between benign remnant tissue and malignancy. Refinement of TgAb assays, with epitope mapping and better standardization, could enhance their value as surrogate markers. Beyond proteins, liquid biopsy technologies, particularly cfDNA, ctDNA, and miRNA assays with fragmentomic or methylation profiling, are being explored as non-invasive tools to monitor disease burden and molecular evolution in DTC. Integration of all these markers into multi-omics strategies may ultimately provide a more robust and individualized follow-up paradigm.

In conclusion, Tg and TgAbs remain essential tools for laboratory monitoring in differentiated thyroid carcinoma, but their interpretation requires a careful understanding of assay performance, analytical limitations, cut-offs, and antibody dynamics. Because these nuances cannot be separated from the patient’s clinical presentation, effective collaboration between clinicians and laboratory specialists is indispensable. Such collaboration ensures that analytical findings are interpreted appropriately and that follow-up decisions truly reflect the patient’s situation. Ongoing technological innovation holds the promise of complementing, or even redefining, the biochemical monitoring of thyroid cancer in the coming years.

## Declaration of interest

The authors declare that there is no conflict of interest that could be perceived as prejudicing the impartiality of the work reported.

## Funding

This research did not receive any specific grant from any funding agency in the public, commercial, or not-for-profit sector.
